# Using local clinical and microbiological data to develop an institution specific carbapenem-sparing strategy in sepsis: a nested case-control study

**DOI:** 10.1186/s13756-019-0465-y

**Published:** 2019-01-25

**Authors:** Merel M. C. Lambregts, Bart J. C. Hendriks, Leo G. Visser, Sandra T. Bernards, Mark G. J. de Boer

**Affiliations:** 1 Department of Infectious Diseases, Leiden University Medical Center, Albinusdreef 2, 2333 RC, Leiden, The Netherlands; 2 Department of Clinical Pharmacy and Toxicology, Leiden University Medical Center, Leiden, The Netherlands; 3 Department of Medical Microbiology, Leiden University Medical Center, Leiden, The Netherlands

**Keywords:** Antibiotic stewardship, Guideline-development, Empiric therapy, Sepsis, Antimicrobial resistance, Gram-negative bacteremia

## Abstract

**Background:**

From a stewardship perspective it is recommended that antibiotic guidelines are adjusted to the local setting, accounting for the local epidemiology of pathogens. In many settings the prevalence of Gram-negative pathogens with resistance to empiric sepsis therapy is increasing. How and when to escalate standard sepsis therapy to a reserve antimicrobial agent, is a recurrent dilemma. The study objective was to develop decision strategies for empiric sepsis therapy based on local microbiological and clinical data, and estimate the number needed to treat with a carbapenem to avoid mismatch of empiric therapy in one patient (NNTC).

**Methods:**

We performed a nested case control study in patients (> 18 years) with Gram-negative bacteremia in 2013–2016. Cases were defined as patients with Gram-negative bacteremia with in vitro resistance to the combination 2nd generation cephalosporin AND aminoglycoside (C-2GC + AG). Control patients had Gram-negative bacteremia with in vitro susceptibility to cefuroxime AND/OR gentamicin, 1:2 ratio. Univariate and multivariable analysis was performed for demographic and clinical predictors of resistance. The adequacy rates of empiric therapy and the NNTC were estimated for different strategies.

**Results:**

The cohort consisted of 486 episodes of Gram-negative bacteremia in 450 patients. Median age was 66 years (IQR 56–74). In vitro resistance to C-2GC + AG was present in 44 patients (8.8%). Independent predictors for resistance to empiric sepsis therapy were hematologic malignancy (adjusted OR 4.09, 95%CI 1.43–11.62, *p* < 0.01), previously cultured drug resistant pathogen (adjusted OR 3.72. 95%CI 1.72–8.03, p < 0.01) and antibiotic therapy during the preceding 2 months (adjusted OR 12.5 4.08–38.48, p < 0.01)**.** With risk-based strategies, an adequacy rate of empiric therapy of 95.2–99.3% could be achieved. Compared to treating all patients with a carbapenem, the NNTC could be reduced by 82.8% (95%CI 78.5–87.5%) using the targeted approaches.

**Conclusions:**

A risk-based approach in empiric sepsis therapy has the potential to better target the use of reserve antimicrobial agents aimed at multi-resistant Gram-negative pathogens. A structured evaluation of the expected antimicrobial consumption and antibiotic adequacy rates is essential to be able to weigh the costs and benefits of potential antibiotic strategies and select the most appropriate approach.

**Electronic supplementary material:**

The online version of this article (10.1186/s13756-019-0465-y) contains supplementary material, which is available to authorized users.

## Introduction

Current guidelines on antibiotic stewardship recommend to adapt empiric therapy to local microbiological data [1]. However, specific recommendations on when and how to change the empiric treatment guidelines in response to increasing resistance rates are lacking. The empiric strategy may need to be broadened to guarantee coverage of the most common pathogens. The downside of this action is an increase in selective pressure, driving further emergence of resistance [2]. Therefore, whether or not to escalate empiric treatment guidelines in response to new resistance data is a recurrent dilemma in antibiotic policy committees all over the world. Strategies that break the vicious circle of increasing resistance and increasing antibiotic consumption are needed [3–5]. The use of a risk-based discrimination in empiric therapy has this potential. If patients with a high probability of infection with a resistant pathogen can be identified upfront, empiric therapy can be escalated selectively [6, 7]. This approach combines the two major aims of antibiotic stewardship: promoting effective antimicrobial therapy in all patients, while limiting antibiotic usage where possible [8]. Both aims are especially relevant in sepsis guidelines [9]. The importance of prompt initiation of effective empiric therapy in this patient category is well recognized [10–14], and the antibiotic consumption associated with empiric treatment for (presumed) sepsis is substantial [15, 16].

In the Netherlands and other countries with low to moderate resistance rates, the standard treatment for sepsis of unknown origin often is a second or third generation cephalosporin (2GC or 3GC) combined with an aminoglycoside (AG) [17]. The prevalence of Gram-negative pathogens that are resistant to this empiric treatment combination, due to production of extended spectrum β-lactamases (ESBL) and other mechanisms, is increasing [18]. This development warrants regular re-evaluation of empiric sepsis therapy recommendations and consideration of escalation to a carbapenem.

The study objective was to explore a practical method to design institutional strategies for empiric therapy based on local microbiological and clinical data, and to estimate the potential treatment adequacy rates and reserve antimicrobial consumption for each of these strategies.

## Methods

The study was conducted according to the approach described in Table 1. This 7-step method is illustrated using local data. The risk factors for bloodstream infection with a Gram-negative organism with reduced susceptibility to standard sepsis treatment were identified in the case-control study. The effect of different targeted empiric therapy approaches on the proportion of patients that receive adequate empiric treatment and the number of patients needed to treat with a carbapenem to avoid mismatch of empiric therapy in one patient (NNTC), were estimated applying the case control study (2013–2016) and the cohort data (2013–2014). The reporting of the results was performed in accordance with STROBE guidelines for cohort and case-control studies. [19]

**Table 1:** 7-step method for the development on institution specific empiric treatment guidelines

	Description	Example
Step 1 The clinical question	Define A) the clinical syndrome for which empiric treatment is re-evaluated, B) the patient population and C) the current empiric treatment guideline.	The clinical syndrome is sepsis. The target patient population is adult patients in an academic medical center. The current empiric treatment for sepsis is C-2GC-AG.
Step 2 Susceptibility data	Determine the local prevalence of resistance to the current empiric treatment (syndrome and population specific).	Of all patients with suspected sepsis, 6.7% are diagnosed with Gram-negative bacteremia.* Gram-negative resistance for C-2GC-AG in blood culture isolates is 8.8%. In the study center. Methicillin resistant *Staphylococcus aureus* (MRSA) and penicillin resistant pneumococcal species are very rare in the Netherlands.
Step 3 Definition of risk factors	Identify available predictors for resistance to the current empiric treatment.	Independent risk factors of resistance to empiric sepsis therapy in the study population are prior antimicrobial use and prior isolates with a DRP.
Step 4 Targeted strategies	Identify potential targeted treatment strategies.	Option A: A carbapenem in patients with a DRP cultured the previous 6 months and C-2GC-AG in other patients. Option B: a carbapenem in all patients with sepsis.
Step 5 Estimating benefit	Estimate the proportion of patients that would be adequately treated if empiric sepsis therapy was changed.	Option A: 95.2% of Gram-negative bloodstream infections would be treated adequately. Option B: 99.8% of Gram-negative bloodstream infections would be treated adequately.
Step 6 Estimating costs	Identify the number needed to treat (NNTC).	Option A: NNTC is 42 patients. Option B: NNTC is 173 patients.
Step 7 Selection of empiric treatment strategy	Balance the cost and benefits of phase 5 and 6 to select the most appropriate strategy.	A moral deliberation with stakeholders was performed to decide on the most appropriate antibiotic therapy for sepsis in the institution. Option A was selected.
Implementation and evaluation	Evaluate the costs and benefits of the selected approach.	After implementation of strategy A, adequacy rates, outcome, side-effects of antimicrobials and antimicrobial consumption were evaluated.

Legend: NNTC = number of patients needed to treat with a carbapenem instead of cefuroxime/gentamicin to prevent one case of inappropriate empiric therapy, C-2GC-AG = cefuroxime combined with gentamicin, DRP = Drug resistant pathogen. * To estimate the overall blood culture positivity rate, the proportion of bacteremia was determined during two separate months, June and December 2014. During this period, all patients in whom blood cultures were obtained because of fever were included. In this pilot period, of all patients with suspected infection, 53/778 (6.7%) had positive blood cultures with a Gram-negative pathogen. All other data used in the example provided in column 3 are cohort data

### Setting and patient population

The study period was defined as from January 2013 to December 2016. The Leiden University Medical Center (LUMC) is a tertiary care hospital in the Netherlands. Standard empiric sepsis therapy in the institution consisted of a second generation cephalosporin, cefuroxime, combined with gentamicin (C-2GC + AG). In 2013–2014, all patients > 18 years of age, with monomicrobial Gram-negative bacteremia were included (cohort 2013–2014). Both community acquired and nosocomial episodes were eligible for inclusion. Patients were identified through search of the microbiology laboratory database. Gram-negative bacteremia was defined as one or more positive blood cultures with a Gram-negative micro-organism. Cases were defined as adult patients with bacteremia with Gram-negative micro-organisms with reduced susceptibility to C-2GC + AG. Reduced susceptibility was defined as intermediate sensitivity (I) or resistance (R) according to the European Committee on Antimicrobial Susceptibility Testing (EUCAST) criteria to 2GC and AG. Control patients were defined as patients with Gram-negative bacteremia with a micro-organism susceptible to 2GC, AG or both. Two control patients per case patient were randomly selected from the cohort. Using the patient identification code, every third patient meeting the criteria for control was selected.

The inclusion period for the case selection was prolonged with two additional years (2013–2016) compared to the cohort (2013–2014), because of the relatively low incidence of combined 2GC and AG resistance. It was assumed that the characteristics of the control and case populations were not variable over the period of study.

### Clinical data

Clinical data were collected from the electronic medical records and included demographics, co-morbidities, clinical characteristics at the time of presentation and known risk factors of antimicrobial resistance [6, 8, 20–23] such as a history of recurrent urinary tract infections (UTI’s), previous hospital stays and previous antibiotic treatment. Previous antibiotic treatment was defined as administration of one or more antibiotic doses during the previous 2 months. Current antibiotic use was defined as at least one administration of antibiotics during the 24 h preceding the collection of blood specimens. For in-hospital and outpatient clinic prescriptions these data were obtained from the institutional electronic prescription system. For other prescriptions, the documented patient history, referral letters and correspondence with other health care providers were searched. Prior known colonization or infection with a drug resistant pathogen (prior-DRP) was defined as the isolation of one of the following pathogens from any body site, including rectal swabs: vancomycin resistant enterococci, methicillin resistant *Staphylococcus aureus*, Enterobacteriaceae with in vitro resistance to AG, second and/or third generation cephalosporins and/or quinolones, *Pseudomonas aeruginosa* with resistance to third generation cephalosporins, AG or quinolones.

In clinical practice, physicians may defer from standard sepsis therapy for a variety of reasons, including a high suspicion of antimicrobial resistance. To assess current practice, the antibiotics that constituted the initial empiric therapy were extracted from the patient records. Empiric therapy was considered adequate if at least one of the antibiotics matched the in vitro susceptibility of the isolated pathogen. Multiple episodes of bacteremia per patient were allowed if the antimicrobial therapy for the previous episode had been completed and clinical and microbiological cure had been achieved.

### Microbiological data

Microbiological data were retrieved from the database of the Microbiology department and included the isolated micro-organism and susceptibility patterns of the current and previous episodes. Blood cultures were incubated using the BACTEC™ blood culture system (Becton Dickinson Benelux, Erembodegem, Belgium). Identification of isolates was performed using matrix-assisted laser desorption/ionisation-time of flight spectrometry (MALDI-TOF) using the Microflex system (Bruker, Bremen, Germany). Antimicrobial susceptibility testing was performed with the VITEK2 system and E-tests (BioMérieux, Brussels, Belgium). Extended-spectrum beta-lactamase (ESBL) production was determined by the use of the combination disc diffusion test [23]. Minimum inhibitory concentration (MIC) breakpoints for resistance and intermediate sensitivity were based on EUCAST criteria [24].

### Statistical analysis

Imputation for missing data was not applied. Categorical variables were reported as counts and percentages and continuous variables as medians with interquartile ranges (IQR). Univariate analysis of clinical predictors of reduced susceptibility to empiric therapy was performed using the Fisher’s exact test and reported as odds ratios (OR) with 95% confidence interval (95% CI). All variables that showed a trend towards an association (*P* < 0.2) were included in the logistic regression analysis. Potential targeted empiric treatment strategies were designed based on the strongest independent predictors of resistance to C-2GC + AG. The proportion of patients with bacteremia that would receive adequate treatment with the strategy (adequacy rate) and the number of patients needed to treat with a carbapenem to avoid mismatch of therapy in one patient (NNTC) were estimated using the formula described in Additional file 1: Supplement A. The data for these estimations were derived from the study cohort: The frequency of the strategies risk factor(s) (cohort 2013/2014), the frequency of reduced susceptibility to gentamicin/cefuroxime and to carbapenems (cohort 2013/2014), and the sensitivity of the specific risk-based strategy for the presence of resistance to cefuroxime/gentamicin (cases 2013–2016). The NNTCs of the risk-based strategies were compared to the theoretical scenario of uniform application of the local sepsis guideline and the actual clinical practice data. The NNTC was assessed for different theoretical probabilities of Gram-negative bacteremia in patients treated empirically for presumed sepsis. All statistical analyses were performed with IBM SPSS Statistics, version 23.

## Results

The cohort (2013–2014) consisted of 486 episodes of Gram-negative aerobic bacteremia in 450 patients. The final database had < 2% missing data. Median age was 66 years (IQR 56–73), in 263 (54.1%) episodes, the patient was male. In this cohort in vitro reduced susceptibility to 2GC monotherapy was present in 176 patients (36.2%), reduced susceptibility to AG in 84 patients (12.6%) and to the combination C-2GC + AG in 43 patients (8.8%). In 95/486 (19.5%) a drug resistant pathogen (DRP) was cultured previously, in 54/95 (56.8%) the prior-DRP was isolated during the preceding 6 months. A total of 144/486 (29.6%) patients were already on antibiotic therapy when they were evaluated for suspected sepsis and 257/486 patients (52.9%) had been treated with antibiotics in the preceding 2 months. Empiric therapy contained a carbapenem in 27/486 (5.6%) of patients. Of the 43/486 (8.8%) patients with in vitro resistance to C-2GC + AG, 12/43 (27.9%) received adequate empiric treatment. The 30-day mortality rate for the cohort was 59/486 (12.1%). Resistance to carbapenems was 1/486 (0.2%).

After applying the case criterion for Gram-negative bacteremia with in vitro reduced susceptibility to cefuroxime and gentamicin, 71 patients (2013–2016) were identified as cases and 142 controls were randomly selected from the remaining patients in the cohort. The demographic and clinical characteristics of cases and controls are shown in Table 2. Additional file 1: Supplement B depicts the pathogen distribution. The causative pathogen was ESBL producing in 64.8% (46/71) and 6.3% (9/142) in cases and controls respectively (*p* < 0.001).

**Table 2:** Demographics and clinical characteristics of cases and controls

Characteristic	Cases n (%)	Controls n (%)	*P* Value	OR (95% CI)
Patient demographics				
Male gender	45 (63.4)	80 (56.3)	.38	1.34 (0.75–2.41)
Age > 65	32 (43.7)	73 (51.4)	.31	0.77 (0.44–1.38)
Medical history				
Diabetes mellitus	19 (26.8)	50 (35.2)	.28	0.67 (0.36–1.26)
Corticosteroid therapy (prior 6 months)	32 (45.1)	47 (33.1)	.10	1.66 (0.93–2.97)
Neutropenia	14 (19.7)	9 (6.3)	.005	3.62 (1.49–8.87)
Solid organ transplantation	14 (19.7)	23 (16.2)	.57	1.27 (0.61–2.65)
Hematologic malignancy	18 (25.4)	9 (6.3)	<.001	5.01 (2.12–11.87)
Non-hematologic malignancy	12 (16.9)	33 (23.2)	.37	0.67 (0.32–1.40)
Chronic urologic disorder	13 (18.3)	33 (23.2)	.48	0.74 (0.36–1.52)
Chronic pulmonary disease	7 (9.9)	19 (13.4)	.51	0.71 (0.28–1.77)
Recurrent urinary tract infections	7 (9.9)	14 (9.9)	1.00	1.00 (0.38–2.60)
Clinical presentation				
Fever (temperature > 38.5 °C)	49 (69.0)	104 (73.2)	.31	0.81 (0.43–1.53)
EMV-score < 15	21 (30.6)	29 (20.4)	.23	1.57 (0.81–3.02)
Hypotension^a^	18 (25.4)	23 (16.2)	.14	1.79 (0.89–3.63)
Current antibiotic use^b^	49 (69.0)	37 (26.1)	<.001	6.32 (3.38–11.84)
Antibiotic usage preceding 2 months	67 (94.4)	67 (47.2)	<.001	18.75 (6.49–54.19)
ICU/MCU > 2 days	11 (15.5)	7 (4.9)	.02	3.54 (1.31–9.57)
ICU/MC preceding 6 months	23 (32.4)	16 (11.3)	<.001	3.77 (1.84–7.75)
Hospital stay preceding 6 months	49 (69.0)	65 (45.8)	.001	2.64 (1.45–4.82)
Hospitalization > 5 days	32 (45.1)	28 (19.7)	<.001	3.34 (1.79–6.24)
Prior-DRP^c^	42 (59.2)	27 (19.0)	<.001	6.17 (3.28–11.61)
Source of infection			.06	–
Urinary tract	23 (32.4)	68 (47.9)		
Intra-abdominal tract	22 (31.0)	44 (31.0)		
Respiratory tract	3 (4.3)	9 (6.4)		
Skin/soft tissue	6 (8.6)	4 (2.8)		
Other	7 (9.9)	7 (4.9)		
Unidentified	10 (14.1)	10 (7.0)		

Data are presented as No. (%). *P* values are calculated by Fisher exact test, *p*-value for ‘source of infection’ was calculated by chi-square test. Abbreviations: OR = odds ratio, EMV-score: eye-motor-verbal score. ICU/MCU = intensive care unit / medium care unit. IQR = interquartile range. ^A^ Hypotension = systolic blood pressure < 90 mmHg or requirement for intravenous vasopressor agents. ^B^ ‘Current antibiotic use’ = at least one administration of antibiotics during the 24 h preceding the collection of blood specimens. ^c^ ‘Prior-DRP’ = one of the following drug resistant pathogens isolated from any body site: Vancomycin resistant enterococci, multi resistant *Staphylococcus aureus*, Enterobacteriaceae with in vitro resistance to aminoglycosides, second and/or third generation cephalosporin’s (including ESBL positive Enterobacteriaceae) and/or quinolones, *Pseudomonas aeruginosa* with resistance to third generation cephalosporins, aminoglycosides or quinolones.

### Risk factors for non-susceptibility to empiric therapy

**Fig. 1:**
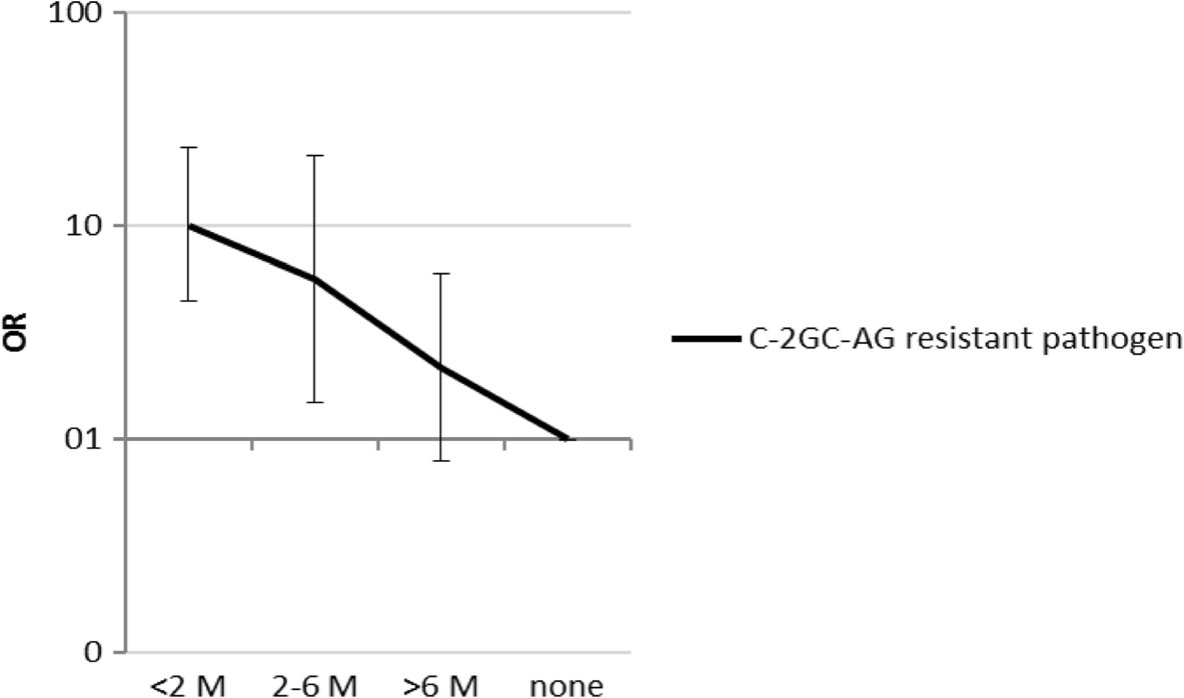
Odds ratio for resistance to empiric therapy related to time since the last drug resistant pathogen (DRP) was cultured. Legend. M = months. C-2GC + AG = Combination 2nd generation cephalosporin and aminoglycoside. Prior-DRP = drug resistant pathogen(s) isolated from any body site: Vancomycin resistant enterococci, multi resistant *Staphylococcus aureus*, Enterobacteriaceae with in vitro resistance to aminoglycosides, second and/or third generation cephalosporin’s (including ESBL positive Enterobacteriaceae) and/or quinolones, *Pseudomonas aeruginosa* with resistance to third generation cephalosporin’s, aminoglycosides or quinolones. Odds ratio for infection with cefuroxime and gentamicin resistant Gram-negative pathogen, for patients with prior-DRP isolated compared to patients without prior-DRP isolates, for different time intervals in months since the last DRP was cultured. Note that the y-axis is on a logarithmic scale

The result of the univariate analyses are shown in Table 2. Patients with hematologic malignancy or neutropenia were at increased risk of a pathogen with reduced susceptibility to C-2GC + AG. Pre-treatment with antibiotics in the 2 months prior to presentation and antibiotic treatment at the day of presentation were associated with presence of reduced susceptibility to C-2GC + AG. In addition, previous admission on general wards, ICU wards and length of hospital stay were strong predictors of reduced susceptibility to standard empiric therapy. The strongest crude predictor was prior isolation of a resistant micro-organism from any site, including rectal swabs. Figure 1 depicts the odds ratio for infection with a pathogen with reduced susceptibility to C-2GC + AG, depending on the time elapsed between the DRP cultures and the current presentation with infection.

In the multivariable analysis a previous culture with a DRP (adjusted OR 3.72 95%CI 1.72–8.03, *p* < 0.01), antibiotic use during the preceding two months (adjusted OR 12.5, 95%CI 4.08–38.48, p < 0.01), and a hematologic malignancy (adjusted OR 4.09, 95%CI 1.43–11.62, p < 0.01) were independently associated with reduced susceptibility (Additional file 1: Supplement C).

### Exploring the effect of risk-based sepsis guidelines: Calculated estimations

**Fig. 2:**
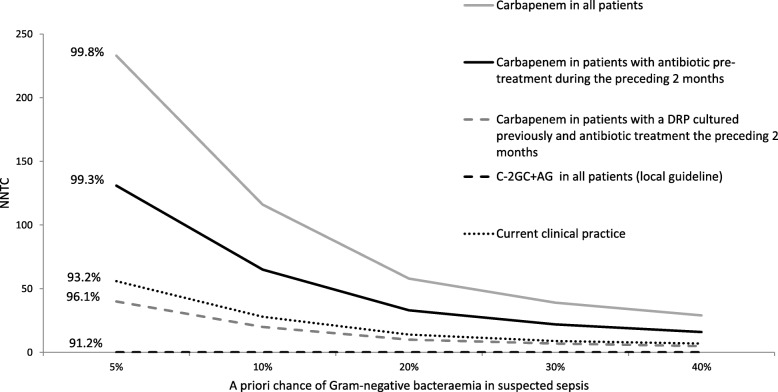
Estimation of the effect of the different empiric strategies on effective therapy rate and consumption of carbapenems, differentiated by a priori probability of bacteremia and compared to other strategies for selection of empiric therapy. Legend. NNTC = number of patients needed to treat with a carbapenem instead of cefuroxime/gentamicin to avoid mismatch of empiric therapy in one patient. C-2GC + AG = 2nd generation cephalosporin/aminoglycoside combination therapy. DRP = drug resistant pathogen(s) isolated from any body site: Vancomycin resistant enterococci, multi resistant *Staphylococcus aureus*, enterobacteriaceae with in vitro resistance to aminoglycosides, second and/or third generation cephalosporin’s (including ESBL positive Enterobacteriaceae) and/or quinolones, Pseudomonas aeruginosa with resistance to third generation cephalosporins, aminoglycosides or quinolones.. Current clinical practice: 2GC + AG as standard therapy, escalation to a carbapenem according to judgment of treating physician. The percentages (91.2–99.0%) indicate the proportion of patients with bacteremia that would receive adequate treatment if the strategy was implemented. For example: if all patients were to be treated with a carbapenem, the overall rate of adequate therapy in patients with bacteremia would be 99.0%. In case of an a priory risk of bacteremia of 10%, the corresponding NNTC is 128 patients

The relevant risk factors for resistance to empiric therapy derived from the multivariable analysis were used to design five different risk-based empiric sepsis treatment strategies. The calculated effect of these individual strategies on the proportion of patients with Gram-negative sepsis that would be treated adequately and the corresponding NNTC are shown in Table 3, and for a selection of strategies in Fig. 2. The NNTC is to a large extent dependent on the number of patients that are empirically treated for sepsis. This number is much larger than the number of patients that are eventually diagnosed with Gram-negative bacteremia. To account for these differences in prevalence of Gram-negative bacteremia amongst patients that are empirically treated for presumed sepsis, the NNTC was assessed for different probabilities of Gram-negative bacteremia. (Fig. 2, Table 3).

**Table 3:** Estimated effects of implementation of different empiric sepsis treatments on effective therapy rate and consumption of carbapenems in a population suspected of Gram-negative bacteremia

Treatment strategy	Sensitivity of the criterion for presence of combined resistance*	Proportion of patients with Gram-negative BSI adequately treated	Proportion of patients with Gram-negative BSI treated with carbapenem	Estimated NNTC** with carbapenem according to frequency of Gram-negative bacteremia in suspected sepsis A priori probability of Gram-negative bacteremia in suspected sepsis ^a^				
				5%	10%	20%	30%	40%
1. Cefuroxime/gentamicin in all patients with sepsis	0	.912	0	–	–	–	–	–
2. Carbapenem in all patients with sepsis	1.000	.998	1.000	233	116	58	39	29
3. Only a carbapenem in patients with antibiotic pre-treatment on day of culture.	.690	.971	.296	100	50	25	17	13
3. Only a carbapenem in patients with antibiotic treatment < 2 months	.943	.993	.529	130	65	33	22	16
4. Only a carbapenem in patients with a DRP^b^ cultured < 6 months	.465	.952	.111	55	28	14	9	7
5. Only a carbapenem in patients with a DRP cultured previously (no time restriction)	.592	.963	.195	76	38	19	13	10
7. Only a carbapenem in patients with a DRP previously and antibiotic treatment < 2 months	.549	.961	.101	42	21	11	7	5
8. Current Practice	.225	.931	.056	57	29	14	10	7

Legend ^A^ Frequency of Gram-negative bacteremia as percentage of the total No. of patients with suspected sepsis in whom empiric therapy is started. ^B^ Drug resistant pathogen(s) (DRP) isolated from any body site: Vancomycin resistant enterococci, multi resistant *Staphylococcus aureus*, Enterobacteriaceae with in vitro resistance to aminoglycosides, second and/or third generation cephalosporin’s (including ESBL positive Enterobacteriaceae) and/or quinolones, *Pseudomonas aeruginosa* with resistance to third generation cephalosporins, aminoglycosides or quinolones.* The sensitivity was derived from the study data (cases 2013–2016) ** NNTC = Number needed to treat with carbapenem instead of cefuroxime/gentamicin to avoid mismatch of empiric therapy for Gram-negative bacteremia in one patient. For the calculation of the NNTC the formula in Additional file 1: Supplement A was applied

Example, strategy 5: Standard empiric treatment is cefuroxime/gentamicin, carbapenems are reserved for patients with a history of drug resistant pathogen (DRP). This results in prescription of a carbapenem in 19.5% of patients with Gram-negative bacteremia. With this strategy, empiric treatment of patients with cefuroxime/gentamicin resistant bacteremia is adequate in 59.2% and the overall treatment adequacy rate in Gram-negative bacteremia is 96.3%. In the scenario of a pre-test probability of Gram-negative bacteremia of 10%, 38 patients would be treated with a carbapenem to avoid mismatch of empiric therapy for Gram-negative bacteremia in 1 patient

In the scenario of ‘standard empiric carbapenem therapy in all patients’, the adequacy rate of empiric therapy was 99.8%. The corresponding NNTC was 29 to 233, depending on the probability (i.e. high: 40% to low: 5%) of Gram-negative bacteremia. Alternatively, risk-based strategies resulted in an estimated adequacy rate of 95.2–99.3%. Compared to treating all patients with a carbapenem empirically, the NNTC in the targeted approaches was a factor 2.3 to 4.6 lower, depending on the selected approach. The NNTC was lowest if a carbapenem would be reserved for patients in whom a DRP was cultured previously and antibiotic treatment had been administered in the preceding 2 months. The estimated reduction of carbapenem use was 82.8% (95%CI 78.5–87.5%). This strategy had a treatment adequacy rate of 96.1% of patients with Gram-negative bacteremia. This is an absolute increase in adequacy rate of 4.9% compared to the local guideline and an absolute increase of 3.0% compared to clinical practice (Fig. 2, Table 3).

## Discussion

Using real-life clinical and microbiological data, we propose a method to develop risk-based empiric antibiotic policies and to estimate the potential costs and benefits of policy changes (Table 1).

Although there are multiple previous prediction rules for infection with resistant pathogens, the applicability of these rules to the selection of institutional empiric antimicrobial treatment is limited. The majority of prediction score studies focused on a specific pathogen or a specific mechanism of resistance, for example ESBL [6, 20, 25–27]. For clinical practice, it is more relevant to predict susceptibility to an empiric regimen in a predefined clinical syndrome, instead of predicting the presence of a specific mechanism of resistance. Secondly, the consequences of implementation of the prediction scores on adequacy rate and/or NNTC are frequently lacking [6, 7]. Thirdly, the susceptibility of pathogens and the risk factors for resistance may vary substantially amongst institutions, making it is necessary to base empiric treatment recommendations on local epidemiology. Our 7-step method can be used to develop institutional empiric policy for a variety of clinical syndromes, and focusses on applicability of the results in daily clinical practice.

In response to increasing resistance rates, we applied the method to improve empiric coverage of causative Gram-negative micro-organisms in sepsis, while maintaining a responsible antimicrobial policy with regard to antibiotic consumption. Our data show that in current practice, clinicians already incorporate an assessment of the risk of a resistant pathogen in decision-making, with a relatively low NNTC. The treatment adequacy rate however, can be further increased using targeted strategies, without increasing inappropriate reserve antimicrobial consumption.

The NNTC was stratified according to the theoretical probability of Gram-negative bacteremia. Previous literature on positivity rates in consecutive blood cultures, shows probabilities of Gram-negative bacteremia below 5% [28, 29]. However, the positivity rate varies substantially depending on the patient population, to up to 41% in septic shock [28–34]. As a result, the NNTC in the critically ill is considerably lower than in a low acuity population [16, 29]. The strategies were based on bacteremia. Including non-bacteremic infections, would further decrease the NNTC. We focused on bacteremia, as the importance of adequate empiric treatment is higher in bacteremic, compared to non-bacteremic episodes.

A limitation of the study is the retrospective data collection. There is potential underreporting of antibiotic pre-treatment. However, this effect is limited, given the use of electronic prescription systems. In addition, potentially important predictive factors, such as travel history, may have been missed, because of limited availability of specific information in the medical charts. Incorporating more determinants, could improve the strategies and further reduce NNTC.

A second limitation is that, in our analysis of the NNTC, we assumed that the identified predictors of antimicrobial resistance are independent of the a priori risk of Gram-negative bacteremia. On theoretical grounds, we do not expect previous antibiotic use and colonization with DRP’s to have an important etiologic effect on the a priori risk of Gram-negative bacteremia itself.

Thirdly, the inclusion period for cases was prolonged compared to the initial cohort, because of the low incidence of C-2GC + AG resistance. Although the epidemiology of antimicrobial resistance is subject to change over time, it is unlikely that the prolonged inclusion period would affect risk factors associated with C-2GC + AG resistance (step 3).

The reported results on Gram-negative bacteremia are institution specific. Differences in antimicrobial susceptibility rates, patient population and treatment guidelines between institutions may all affect treatment adequacy rates and the NNTC. However, the method that was used to determine a center-specific NNTC is applicable in every setting.

From a scientific perspective, prospective validation within the institution is preferable, before implementation is considered. However, prospective validation would hamper a timely response to the latest resistance data, resulting in a difficult process of catch-up because of changing epidemiology. Therefore, cyclic evaluation and optimization within the institution after implementation is - from a practical point of view - preferable to further improve targeted antibiotic strategies.

In step 7, the benefits of adequate therapy and the costs of the associated antimicrobial consumption need to be weighed to select the most appropriate strategy. The rate of inadequate empiric therapy that clinicians are willing to accept, varies according to the severity of the clinical syndrome. For sepsis, and especially septic shock, the optimal balance between antibiotic adequacy rate and consumption of reserve antimicrobial agents is incomparable to the setting of more benign infections, for example cystitis. How to balance these aspects is highly complex. This also involves ethics, as decisions do not merely affect patients today, but impacts future generations as well [35]. The number needed to treat with reserve antimicrobial agents contributes to this ethical discussion. This study demonstrates the feasibility of generating these numbers for the local situation.

## Conclusions

The present study exemplifies a method to develop risk-based empiric antibiotic policies and estimate the effects on treatment adequacy and antimicrobial consumption. The approach has the potential to target the use of reserve antimicrobial agents and can be applied in different clinical settings to optimize empiric antibiotic therapy.

## Additional file

Additional file 1: **Supplement A.** Formula for the estimation of the number needed to treat with a carbapenem. **Supplement B.** Isolated pathogens in cases (n=71) and controls (n=142). **Supplement C.** Multivariable analysis of predictors of infection with a pathogen with reduced susceptibility to treatment with cefuroxime and gentamicin. (DOCX 20 kb)
