# Supplementary material for: Using local clinical and microbiological data to develop an institution specific carbapenem-sparing strategy in sepsis: a nested case-control study
Source: Antimicrob Resist Infect Control. 2019 Jan 25;8:19. doi: 10.1186/s13756-019-0465-y (PMC6347774; doi:10.1186/s13756-019-0465-y)
Supplement: Supplementary file 1 — Supplement A. Formula for the estimation of the number needed to treat with a carbapenem. Supplement B. Isolated pathogens in cases (n=71) and controls (n=142). Supplement C. Multivariable analysis of predictors of infection with a pathogen with reduced susceptibility to treatment with cefuroxime and gentamicin. (DOCX 20 kb) [file 13756_2019_465_MOESM1_ESM.docx]

**Supplement A.** Formula for the estimation of the number needed to treat with a carbapenem.

$$NNTC=\frac{\mathrm{PropRf}}{(PropRx-PropRy) \times SensRF}\times\frac{1}{\mathrm{Gramnegbac}}$$

*NNTC*= Number needed to treat with a carbapenem instead of cefuroxime/gentamicin to avoid mismatch of therapy in one patient.

*PropRf* = The frequency of cases with the risk factor (or risk factor combination) as a proportion of the total No. of cases in the study cohort “Gram-negative bacteremia”.

$PropRx$ = The frequency of cases with a pathogen that has reduced susceptibility to the combination therapy gentamicin and cefuroxime (C-2GC+AG) as a proportion of the total No. of cases in the study cohort “Gram-negative bacteremia” .

*PropRy* = The frequency of cases with a pathogen with reduced susceptibility to carbapenems as a proportion of the total No. of cases in the study cohort “Gram-negative bacteremia” .

*SensitivityRF* = Sensitivity of the risk factor (or risk factor combination) for combined resistance to gentamicin and cefuroxime in patients with bloodstream infection with a pathogen with reduces susceptibility to C-2GC+AG.

*Gramnegbac:* A priori probability of Gram-negative bacteremia in suspected sepsis: The frequency of Gram-negative bacteremia as a proportion of the total No. of patients with suspected sepsis in whom empiric therapy is started.

**Example**:

In the study cohort, the resistance rate to the combination cefuroxime/gentamicin was 8.8%. In this cohort, a drug resistant pathogen (DRP) was diagnosed the previous 6 months in 11.1% of cases. Of all patients with bacteremia with a pathogen with reduced susceptibility to C-2GC+AG in 45,5% a drug resistant pathogen was isolated the preceding 6 months. In the study center 6.7 percent of patients in whom blood cultures are obtained are diagnosed with Gram-negative bacteremia.

$$NNTC=\frac{0.111}{(0.088-0.002) \times0.0.465}\times\frac{1}{0.067}=42$$

The number needed to treat with a carbapenem instead of cefuroxime/gentamicin to treat one patient adequately = 42.

**Supplement B.** Isolated pathogens in cases (n=71) and controls (n=142)

|  | **Cases**  **n (%)** | **Controls**  **n (%)** | **p-value*** |
| --- | --- | --- | --- |
| **Pathogen** |  |  | .12 |
| *Escherichia coli* | 34 (47.9) | 83 (58.5) |  |
| *Klebsiella species* | 13 (18.3) | 25 (17.6) |  |
| *Pseudomonas aeruginosa* | 9 (12.7) | 11 (7.7) |  |
| *Serratia marcescens* | 7 (9.9) | 9 (6.3) |  |
| Other Gram-negative pathogens** | 8 (11.3) | 14 (9.9) |  |

**Legend**: *p-value calculated by Fisher exact test. ** *Citrobacter spp*, *Enterobacter spp*,

*Proteus spp*, *Morganella spp* and *Providencia spp*.

**Supplement C.** Multivariable analysis of predictors of infection with a pathogen with reduced susceptibility to treatment with cefuroxime and gentamicin.

|  | **OR** | | **95% CI** | | **p-value** | |  |
| --- | --- | --- | --- | --- | --- | --- | --- |
| Hematologic malignancy | 4.09 | | 1.43-11.62 | | <0.01 | |  |
| Admitted to IC/MC unit ≥ 2 days | | 1.25 | | 0.38-4.12 | | 0.72 | |
| Hospital stay during the preceding 6 months | | 0.94 | | 0.44-2.04 | | 0.88 | |
| Current hospital stay ≥ 5days | | 1.05 | | 0.45-2.42 | | 0.92 | |
| Prior-DRP | | 3.72 | | 1.72-8.03 | | <0.01 | |
| Antibiotic therapy during preceding 2 months | | 12.5 | | 4.08-38.48 | | <0.01 | |

**Legend**. Logistic regression analysis. OR = Adjusted odds ratio, 95%CI = 95% confidence interval. IC/MC = intensive care/medium care. Prior-DRP = Drug resistant pathogen, defined as the isolation of one of the following pathogens from any body site, including rectal swabs: vancomycin resistant enterococci, methicillin resistant *Staphylococcus aureus*, Enterobacteriaceae with in vitro resistance to aminoglycosides, second and/or third generation cephalosporins and/or quinolones, *Pseudomonas aeruginosa* with resistance to third generation cephalosporin’s, aminoglycosides or quinolones.
